# Joint attention performance in preschool-aged boys with autism or fragile X syndrome

**DOI:** 10.3389/fpsyg.2022.918181

**Published:** 2022-08-08

**Authors:** Angela John Thurman, Amanda Dimachkie Nunnally

**Affiliations:** ^1^Department of Psychiatry and Behavioral Sciences, University of California, Davis, Sacramento, CA, United States; ^2^Medical Investigation of Neurodevelopmental Disorders (MIND) Institute, University of California, Davis, Sacramento, CA, United States

**Keywords:** autism spectrum disorder, fragile X syndrome, joint attention, language, anxiety

## Abstract

Early development marks a period of rapid learning facilitated by children’s natural curiosity about the people around them. In children with typical development, these early social attentional preferences set the foundation for learning about and from the surrounding world of people. Much of this learning happens using joint attention, the ability to coordinate attention between people and objects of mutual interest. It is well documented that decreased gaze use is commonly observed in individuals with autism and individuals with fragile X syndrome (FXS). Despite the growing body of research comparing phenotypic similarities between individuals with autism and individuals with FXS, no studies have directly compared joint attention performance between these groups. In the present study, we considered the similarities and differences in joint attention between preschool-aged boys with autism or FXS, and the relation between joint attention, language, and other phenotypic characteristics known to differ between boys with autism and boys with FXS. Although joint attention appeared similar, between-group differences emerged when controlling for the influence of age, non-verbal IQ, and autism symptom severity. Differences were also observed when considering how joint attention performance related to other aspects of the phenotype. For example, strong positive associations were observed between joint attention and language performance in boys with autism but not boys with FXS, even after controlling for non-verbal IQ. In contrast, the negative association between joint attention and anxiety symptom severity was significant and stronger in boys with FXS than in autism. These data offer preliminary insights into the similarities and differences between the autism and FXS phenotypes.

## Introduction

Social attention plays a pivotal role in children’s early learning. For example, the ability to coordinate attention between a social partner and objects or events of mutual interest, known as joint attention (JA), is crucial for socio-cognitive development (Morales et al., 2000; Adamson et al., 2009; Mundy and Bullen, 2022). The development of JA skills may reflect the maturation of socio-cognitive and attentional capabilities that facilitate language learning (Mundy and Gomes, 1998). By adopting a shared frame of reference, JA skills allow children to participate in social learning situations that facilitate mapping words onto their intended referents (Morales et al., 2000; Adamson et al., 2009). Early challenges in JA may create a developmental cascade altering children’s early language development (Mundy and Bullen, 2022). In the present study, we considered JA performance in boys with autism and boys with fragile X syndrome (FXS). Despite the growing body of research comparing the autism and FXS phenotypes, direct comparisons between JA skills across phenotypes are limited. Understanding how JA skills compare between boys with autism and boys with FXS and the associations between JA and other developmental characteristics will aid our understanding of the mechanisms underlying both phenotypes.

### Joint attention and autism symptomatology

JA delays are among the earliest emerging behavioral features associated with autism (Werner et al., 2005). Moreover, in individuals with autism, early JA skills have been shown to predict autism symptomatology in adulthood (Gillespie-Lynch et al., 2012). These early delays in JA development are considered a critical indicator of, and contributor to, a modified trajectory of social learning in individuals with autism (Mundy and Bullen, 2022). Indeed, children with autism display fewer JA acts than children with typical development and children with other developmental disabilities (Wetherby and Prutting, 1984; Loveland and Landry, 1986; Mundy et al., 1986; Dawson et al., 1998; Stallworthy et al., 2022). Moreover, JA delays in children with autism are observable by 9 months of age (Chawarska et al., 2013; Gangi et al., 2016; Stallworthy et al., 2022). Because JA difficulties are among the earliest emerging features associated with the autism phenotype, considerations of JA skills are found in diagnostic and screening assessments of autism symptomatology (Constantino and Gruber, 2012; Lord et al., 2012).

Autism symptomatology is also a hallmark feature of the FXS phenotype. FXS is the most common monogenetic cause of autism and results from a trinucleotide (CGG) expansion in the *FMR1* gene, located on the X chromosome (Oostra and Willemsen, 2003). Because females with FXS have a second, unaffected X chromosome, which can continue to serve as a protective factor, the FXS is more pronounced in males (Tassone et al., 1999; Loesch et al., 2004; Stembalska et al., 2016). Indeed, nearly all males with FXS demonstrate symptoms consistent with the autism phenotype, and 60% or more meet the criteria for an autism diagnosis when using gold-standard assessment tools (Clifford et al., 2007; Harris et al., 2008; Kaufmann et al., 2008; Abbeduto and McDuffie, 2010; Budimirovic and Kaufmann, 2011; Klusek et al., 2014). Although there are numerous similarities between the autism and FXS phenotypes, significant differences are also observed. At the group level, autism symptomatology is milder in FXS than in autism, even when only considering children with FXS who meet diagnostic criteria for ASD (Wolff et al., 2012; McDuffie et al., 2015). Differences have also been observed across groups in the developmental features correlated with autism symptomatology (Thurman et al., 2015). These subtle differences may significantly impact the developmental trajectories associated with these phenotypes. Outside of comparisons of diagnostic assessments that include items focused on JA performance on the ADOS-2, there are no direct comparisons of JA performance between boys with autism and boys with FXS. Understanding the similarities and differences in JA performance can provide essential insights into these phenotypes’ developmental mechanisms.

### Language difficulties associated with autism or fragile X syndrome

Delays in language development are common in young children with autism or FXS (De Giacomo and Fombonne, 1998; McDuffie et al., 2017) and are often observed well beyond early childhood (Hartley et al., 2011; Howlin et al., 2014). For individuals with autism, language delays are often noticed early in development (Tager-Flusberg et al., 2009; Boucher, 2012; Talbott et al., 2015). Approximately 30% of individuals with autism demonstrate limited spoken language skills into the school-age years (Tager-Flusberg and Kasari, 2013). Moreover, approximately half for those who do acquire spoken language demonstrate language delays relative to both chronological age or non-verbal cognitive ability expectations (Kjelgaard and Tager-Flusberg, 2001; Boucher, 2012).

Similarly, early expressive language delays are often observed in individuals with FXS (Roberts et al., 2001; Kover et al., 2015), with up to 30% of individuals still demonstrating limited spoken language skills into adolescence (Levy et al., 2006). Moreover, in individuals with FXS, delays in language performance are often more significant than expected relative to achievements in non-verbal cognition; however, there is some variation as a function of language domain and developmental period considered (Abbeduto et al., 2016).

### Association between joint attention and language skills in autism or fragile X syndrome

Because JA delays are among the earliest emerging behavioral features associated with autism (Werner et al., 2005), there is a robust literature considering the association between JA and language performance in children with autism (Bottema-Beutel, 2016). Numerous investigations have demonstrated a significant association between JA and language performance. Some types of JA (e.g., responding to JA) demonstrate lasting and significant associations even after controlling for non-verbal cognitive ability (Charman, 2003; Sigman and McGovern, 2005; Yoder et al., 2015; Bottema-Beutel, 2016). Moreover, interventions targeting JA skills have positively impacted language outcomes, offering additional support for the role of JA in language development (Kasari et al., 2006, 2012).

In FXS, however, few studies have considered JA skills or the association between JA and language performance. Nonetheless, there is reason to posit that children with FXS may demonstrate delays in JA skills. For example, nearly all males with FXS have been found to have limited gaze use (Murphy et al., 2007; Hall et al., 2009), a critical JA skill. Moreover, Hahn et al. (2016) found that joint engagement (i.e., states in which the child is actively engaged with objects and people) during play with a caregiver was indeed positively associated with expressive language performance and negatively associated with autism symptomatology scores.

Finally, data from the limited studies that have directly compared performance between boys with autism and boys with FXS suggest potential between-group differences in JA performance. For example, when considering autism symptomatology between the two groups, Wolff et al. (2012) found that boys with FXS + ASD were less impaired than boys with autism in response to JA, and McDuffie et al. (2015) found that boys with FXS + ASD were less impaired in showing and sharing attention than were boys with autism. In addition, some recent studies have documented strength in language performance (e.g., vocabulary) in boys with FXS relative to boys with autism, particularly when you control for between-group differences in non-verbal cognitive ability (McDuffie et al., 2013; Thurman et al., 2017; Sterling, 2018; Thurman and Hoyos Alvarez, 2020). It is plausible that early between-group differences in JA skills may contribute to some of the strengths in language development observed in boys with FXS. However, there are no direct comparisons between boys with autism and boys with FXS in the associations between JA and language performance.

### Other attention-modifying phenotypic considerations

Notably, other behavioral similarities are observed between the autism and FXS phenotypes in domains that may also impact the development and/or the measurement of JA performance, such as inattention/hyperactivity (Mayes et al., 2012; Thurman et al., 2014) and anxiety (Leyfer et al., 2006; de Bruin et al., 2007; Cordeiro et al., 2011). Consistent with findings in other domains, despite the similarities observed, differences are also noted in the presence of symptoms of inattention/hyperactivity and anxiety. For example, Thurman et al. (2014) found, while controlling for various developmental characteristics, that parent ratings of anxiety and manic/hyperactive behaviors were significantly higher for boys with FXS than for boys with autism. In addition, the authors found that the association between social avoidance and general anxiety was significantly higher for boys with FXS than for boys with autism. Indeed, increased rates of attentional or anxiety symptomatology may modify how children engage with others and, in turn, influence the development or measurement of JA performance (Salley and Colombo, 2016). Considering these developmental differences may reveal whether similar or different developmental mechanisms underlie shared symptomatology between boys with autism and boys with FXS (Thurman et al., 2015).

### Present study

Despite the growing body of research comparing the autism and FXS phenotypes, direct comparisons of JA skills are limited. These comparisons are needed to clarify the associations between JA, attention-modifying phenotypic characteristics, and language. Moreover, such data can provide insights into these phenotypes’ developmental mechanisms. Research comparing JA skills and their associations to language and other attention-modifying phenotypic characteristics can provide insights into the developmental mechanisms underlying these phenotypes. In the present study, we provide a preliminary examination of JA performance in preschool-aged boys with autism or FXS to answer the following questions:

1. Does JA performance differ between preschool-aged boys with autism and boys with FXS?
2. Is JA performance concurrently associated with language ability, specifically vocabulary ability, in boys with autism and boys with FXS after controlling for the influences of non-verbal cognitive ability? Note, because of the age and language delays associated with both phenotypes, the language measures used in the present study focused heavily on vocabulary ability.
3. Is JA performance concurrently associated with other child characteristics, such as autism symptom severity or other attention-modifying behavioral features observed to differ between boys with autism and boys with FXS (i.e., ADHD and anxiety symptomatology)?

## Materials and methods

### Participants

Fifty-one participants between 3.00 and 5.50 years of age were included in the present study, 30 males with autism and 21 males with FXS. Descriptive statistics for the sample are presented in Table 1. Significant between group differences were observed between participants with autism and participants with FXS differed on Non-verbal IQ scores [*t*(1, 49) = 3.35, *p* = 0.002, Cohen’s *d* = 0.95] and on autism symptom severity scores [*t*(1, 49) = 2.13, *p* = 0.04, Cohen’s *d* = 0.62]. For the participants with autism, the racial/ethnic composition of the sample was 25% Hispanic, with 6.7% Asian, 6.7% Black/African American, 63.3% Caucasian, 20.0% Multi-racial, and 3.3% preferring not to answer. For participants with FXS, the racial/ethnic composition of the sample was 19% Hispanic, with 4.8% Black/African American, 71.4% Caucasian, 19% Multi-racial, and 4.8% preferring not to answer.

**TABLE 1:** Descriptive statistics for participant groups.

	Autism (*n* = 30) *M* (*SD*, range)	FXS (*n* = 21) *M* (*SD*, range)
CA	4.37 (0.82, 3.08–5.56)	4.20 (0.83, 3.02–5.55)
Non-verbal cognition (IQ)	78.17 (20.88, 30–113)	59.48 (17.53, 30–91)
Verbal cognition (IQ)	69.30 (21.75, 30–100)	62.62 (20.51, 30–93)
Autism symptom severity	7.07 (1.84, 4–10)	5.79 (2.35, 2–10)
Receptive vocabulary knowledge (growth score)	91.10 (29.74, 12–137)	81.10 (30.11, 12–122)
Expressive vocabulary knowledge (growth score)	101.03 (31.42, 42–150)	85.90 (29.98, 45–129)
Play: # utterances	113.27 (70.76, 0–225)	114.38 (99.24, 0–378)
Play: weighted comm score	164.63 (110.97, 0–367)	162.76 (150.22, 0–565)
ADHD symptomatology (total score)	27.13 (12.20, 7–58)	30.48 (8.64, 15–45)
Anxiety symptomatology (total score)	30.54 (18.30, 9–75)	36.15 (16.04, 2–67)

PPVT-4, Peabody Picture Vocabulary Test-4; EVT-2, Expressive Vocabulary Test-2; DAS-II SNC, Differential Ability Scales-II Special Non-verbal Composite.

Participants were drawn from a longitudinal study focused on elucidating the mechanisms underlying word learning in boys with autism or FXS. Documentation of an existing diagnosis was provided by families of participants with autism (i.e., existing community diagnosis of autism spectrum disorder) and families of participants with FXS (i.e., documentation of a diagnosis of the FMR1 full mutation, with or without mosaicism). Diagnoses for participants with autism were confirmed through administration of the Autism Diagnostic Observation Schedule-2 (Lord et al., 2012). In addition, all participants enrolled in the present study met the following criteria (based on parent report): (a) English is the primary language of exposure; (b) no sensory or physical impairments that would limit participation in project activities; and (c) no medical conditions (e.g., severe and frequent seizures) that prevented them from meeting the demands of the testing protocol.

Multiple sources were utilized for recruitment, including the MIND Institute’s IDDRC Clinical Translational Core Registry, parent listservs, the National Fragile X Foundation, and clinics and preschools specialized in working with children with NDDs. Due to differences in prevalence rates, participants with autism were more likely to reside locally than were those with FXS.

### Methods

Approval from the Institutional Review Board, as well as written informed consent from the parent/guardian of all participants; verbal assent by the child was not required due to the chronological age and developmental delays displayed by the children in the present study. Visit study measures were administered over the course of two consecutive days. All assessments took place in the research laboratory and were conducted by PhD-level study personnel or trainees under their supervision. Multiple direct assessment and caregiver-report measures were utilized in the present study.

#### Joint attention

The child’s ability to coordinate attention between the examiner and an object was examined during a semi-structured play assessment, using the procedures outlined by Thurman and Mervis (2013). Specifically, two sets (version A and version B) of 30 toys/objects were created, each divided into six blocks of five. Four of the five toys/objects in each block were assigned to an elicitation condition in which the child’s gaze behavior was considered following the presentation of a specific gesture made by the examiner (i.e., giving, blocking, teasing, or point/gaze gesture). In each block, elicitation condition was randomized. In addition, the semi-structured play assessment was completed over the course of 2 days in order to minimize testing fatigue and maximize the naturalistic quality of the elicitation conditions. Version was counterbalanced across participants in each group.

The Blocking/Teasing/Giving conditions were based on the goal ambiguity task developed by Phillips et al. (1992), which was designed to assess the child’s used of JA in response to gestures made by an adult, which varied with regard to the ambiguity of the adult’s intention. The Response to JA trials (Carpenter et al., 1998; Brooks and Meltzoff, 2002) were designed to examiner the child’s ability to monitor the adult’s looking/gazing behavior.

(1) Block: Once the child was manually and visually engaged with the toy, the examiner covered the child hands, blocking the child from further activity.
(2) Tease: The examiner offered the child the toy. Once the child reached for the toy, the examiner quickly withdrew the toy out of the child’s reach.
(3) Give: The examiner handed the child the toy and allowed the child to play with it.
(4) Response to JA: Trials were administered during transitions between toys presented to the child. The examiner made eye contact with the child, and once eye contact was established, turned to the images on either the right or left side of the table while demonstrating the appropriate cue (i.e., cross-midline point + gaze following vs. gaze following only) in conjunction with saying, “Oh, wow!).

During each elicitation, the examiner looked at the child (or looked at the object, during response to JA trials) with neutral affect during a 4-s wait period or until the child initiated/responded with JA (whichever came first). Once the 4-s wait-period elapsed (or the child initiated JA), the examiner resumed play (e.g., giving the toy to child or playing with the toys at the table). The child received a point for each trial in which they demonstrated a JA response during the 4-s wait period; a point was not assigned on trials in which the child did not demonstrate a JA response during the wait period. The total score (across all trials) was used to assess JA in the present study. These tasks were selected as a starting point for considering JA performance, because at the time of data collection, the Phillips et al. (1992) Blocking Tasks had been integrated into the ADOS-2 as method of eliciting JA (Lord et al., 2012) and were used to by Thurman and Mervis (2013) to characterize between group differences between children in the same age range with two other neurodevelopmental disabilities associated with varying social communication phenotypes.

#### Language measures

##### Receptive vocabulary knowledge

The Peabody Picture Vocabulary Test–4th Edition (Dunn and Dunn, 2007) was used to assess receptive vocabulary knowledge in the present study. The PPVT-4 is an individually administered assessment designed for children and adults aged 2.5–90 years and older. Two versions of this measures (i.e., Version A and Version B) are available and were alternated across participants in each group; thus, approximately half of the participants in each group received Version A and half of the participants received Version B of this measure. Growth scores were utilized in study analyses.

##### Expressive vocabulary knowledge

The Expressive Vocabulary Test–2nd Edition (Williams, 2007) was used to assess expressive vocabulary knowledge in the present study. The EVT-2 is an individually administered assessment designed for children and adults aged 2.5–90 years and older. Two versions of this measures (i.e., Version A and Version B) are available and were alternated across participants in each group; thus, approximately half of the participants in each group received Version A and half of the participants received Version B of this measure. Growth scores were utilized in study analyses.

##### Naturalistic language sample

The Abbreviated- Communication Play Protocol, a 20-min semi-structured play sample with a caregiver, was used as the naturalistic language sample (Adamson et al., 2009). The CPP-A consists of four 5-min activities in which caregivers press for different communicative functions by modifying how they use each toy set. Samples start with a 5-min free play activity, where the parent plays with the child as they usually would. Three additional communication contexts are considered: (1) Social Interaction—parents engage in turn-taking games with the child; (2) Requesting—toys placed out of child reach; and (3) Commenting—parents share a series of objects with the child (Adamson et al., 2009). During this sample, each occurrence of the child’s single-word utterances and multiple-word utterances were coded using the Behavioral Observation Research Interactive Software (Friard and Gamba, 2016). Utterances were segmented into C-Units, providing an objective criteria for segmenting utterances (Abbeduto et al., 2020). Specifically, at the upper bound, C-units include an independent clause and any of its modifiers; at the lower bound, any sentence fragment and elliptical response also constitutes a C-unit (Thurman et al., in press). The frequency of each utterance was weighted, such that single-word utterances counted as one point and multiple-word utterances counted as two points. The Weighted frequency total and the Unweighted frequency total (i.e., the number of utterances produced without weighting) were considered in the study analyses.

#### Other child characteristics

##### Non-verbal cognition

The Differential Ability Scales-II Upper-Level Early Years (Elliott, 2007) was used to assess non-verbal cognitive ability in the present study. The DAS-II in an individually administered assessment of general intellectual functioning, designed for children aged 2.5–8 years of age. The Special Non-verbal Composite, which reflects non-verbal cognition using both non-verbal reasoning and non-verbal spatial subtests was used in study analyses.

##### Autism symptomatology

The ADOS-2 (Lord et al., 2012) was used to assess autism symptom severity in the present study. The ADOS-2 is a semi-structured observational assessment, administered by a trained examiner, which is designed to press for the social affective and restricted and repetitive behaviors associated with autism. In the present project, 35 participants received a Module 1 (Autism: *n* = 20, FXS: *n* = 15) and 15 participants received a Module 2 (Autism: *n* = 10, FXS: *n* = 5). Data was missing for two participants due to fatigue. All examiners on the project met research reliability training standards.

##### Attention/hyperactivity symptomatology

The ADHD Rating Scale-IV Preschool Version (McGoey et al., 2007), an 18-item caregiver report measure designed to assess attention deficit and hyperactivity disorder symptoms outlined in the Diagnostic and Statistical Manual of Mental Disorders, Fourth Edition, Text Revision (American Psychiatric Association, 2000) ADHD. Items are rated on a scale of 0 (not at all) to 3 (very often); total score was used in study analyses.

##### Anxiety symptomatology

The Revised Preschool Anxiety Scale (Edwards et al., 2010), a 30-item caregiver report measure designed to assess anxiety symptoms associated with social anxiety, separation anxiety, general anxiety, specific fears, and obsessive-compulsive symptoms. Items are rated on a scale from 1 (not at all true) to 4 (very often true); total score was used in study analyses.

### Analysis plan

A series of analyses were used to consider whether JA scores differed between boys with autism and boys with FXS. First, a univariate analysis of variance was used to directly compare JA scores across the two samples. Second, a regression analysis was used to compare performance across the groups, after controlling for the effects, of CA, non-verbal IQ, and overall autism symptom severity. We corrected for multiple comparisons by using Benjamini and Hochberg’s false discovery rate [FDR; 39] procedures in order to maintain a familywise alpha rate of *p* ≤ 0.05. Finally, descriptive statistics (i.e., means and standard errors) regarding the patterns observed across the different JA elicitation contexts are provided.

Parametric correlations were used to evaluate the correlations between total JA scores and performance on the language ability measures, after controlling for the influence of non-verbal cognitive ability. In each of these analyses, the FDR was used to maintain a familywise alpha rate of p ≤ 0.05. Parametric correlations were also used to evaluate the correlations between total JA scores and other child characteristics (i.e., autism symptom severity, ADHD symptomatology, and anxiety symptomatology).

## Results

### Between-group comparisons of joint attention

**FIGURE 1:**
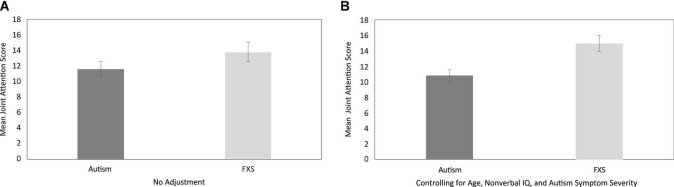
Between-group descriptive comparisons of total joint attention performance, with no adjustment **(A)** and after controlling for the effects of age, non-verbal IQ and autism symptom severity **(B)**.

First, we considered whether JA scores differed between boys with autism and boys with FXS, using a series of analyses. Overall JA scores between boys with autism and boys with FXS were directly compared. Results indicated that mean overall JA scores were slightly higher in boys with FXS (*M* = 14.16, *SD* = 5.75) than in boys with autism (*M* = 11.57, *SD* = 5.10), but the statistical comparison of scores did not reach criterion for a significant between-group effect [*F* (1, 49) = 2.87, *p* = 0.10, partial eta squared = 0.06]. That said, the regression model considering overall JA scores, after controlling for the effects of CA, non-verbal IQ, and overall autism symptom severity, were significant [*F* (4, 48) = 12.76, *p* < 0.001, *R^2^_*adj*_* = 0.54]. Specifically, diagnostic group uniquely accounted for approximately 16% of the variance in overall JA scores, with overall JA scores approximately four points higher for boys with FXS than for boys with autism (*p* = 0.006 and remained significant after FDR correction). See Figure 1 for graphs of JA score comparisons before and after controlling for CA, non-verbal IQ and autism symptom severity.

**FIGURE 2:**
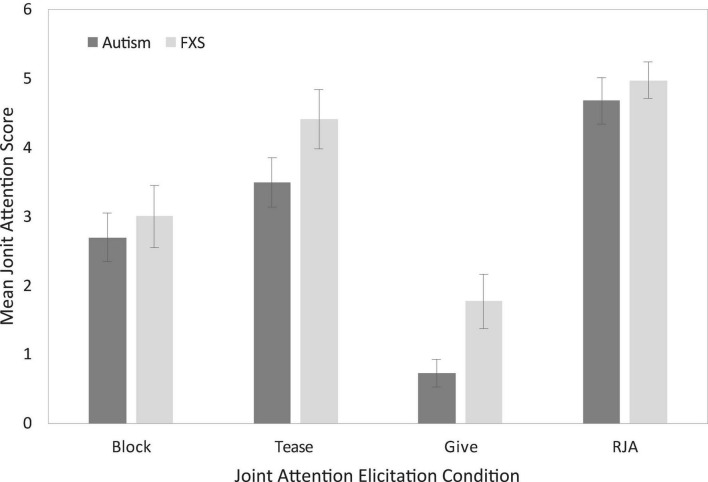
Uncorrected between-group descriptive comparisons of joint attention performance as a function of elicitation condition.

Finally, to provide preliminary data on the patterns observed across JA contexts, we considered participant performance across the different elicitation conditions. Figure 2 presents comparisons of mean JA scores as function of task for boys with autism and boys with FXS. Across all comparisons JA scores were slightly higher for boys with FXS than for boys with autism.

### Association between joint attention and language performance

Analyses were then conducted to consider the relations between JA and language performance for boys with autism and boys with FXS. The contributions of non-verbal cognitive ability were partialed out of this correlation due to its association with both language ability (ASD: *r*s: 0.59–0.70, *p*s < 0.001; FXS: *r*s: 0.55–0.70, *p*s: 0.01–0.004) and JA performance (ASD: *r* = 0.54, *p* = 0.002; FXS: *r* = 0.42, *p* = 0.06). After controlling for the influence of non-verbal cognitive ability, overall JA scores were strongly correlated with all language measures considered in boys with autism, with all associations significant after applying the FDR correction (see Table 2). For boys with FXS, JA scores were not observed to be significantly associated with performance on any of the language measures.

**TABLE 2:** Correlations between joint attention performance and language measures after controlling for non-verbal IQ.

	PPVT-4 growth score	EVT-2 growth score	Play: # utterances	Play: weighted comm score
	**Correlations controlling for DAS-II SNC**			
Autism	0.72***	0.73***	0.67***	0.72***
FXS	0.14	0.20	–0.14	–0.19

***p < 0.001.

PPVT-4, Peabody Picture Vocabulary Test-4; EVT-2, Expressive Vocabulary Test-2; DAS-II SNC, Differential Ability Scales-II Special Non-verbal Composite.

### Relations between joint attention and other child characteristics

Finally, we considered the relations between JA and other child characteristics known to influence social attention (see Table 3). In both boys with autism and boys with FXS, JA performance was negatively associated with autism symptomatology. In boys with autism, but not boys with FXS, JA performance was negatively correlated with ADHD symptomatology. Finally, in boys with FXS, JA performance was negatively associated with anxiety symptomatology in boys with FXS; this association was not significant for boys with autism. All significant correlations remained after applying the FDR correction.

**TABLE 3:** Correlations between joint attention performance and phenotype control measures.

	Autism	FXS
Autism symptoms	–0.39*	–0.61**
ADHD symptoms	–0.44*	–0.11
Anxiety symptoms	0.27	–0.58**

*p < 0.05, **p < 0.01.

DAS-II SNC, Differential Ability Scales-II Special Non-verbal Composite; ADOS-2 Overall CSS, Autism Diagnostic Observation Schedule-2 Overall Comparison Severity Scores.

## Discussion

The goal of the present study was to provide preliminary insights into the similarities and differences in the use of JA skills between boys with autism or FXS and the phenotypic and behavioral characteristics that were concurrently associated with children’s use of JA. Several findings emerged from this study, including group differences in JA performance between boys with autism and those with FXS. In addition, data from the current study suggests that there may be between-group differences in the phenotypic factors associated with JA performance. Altogether, these findings begin to elucidate the different contributors to JA performance in boys with autism and boys with FXS, which has implications for understanding phenotypic differences in the development of JA for these populations.

Although the literature indicates that JA delays are common in children with autism (Wetherby and Prutting, 1984; Loveland and Landry, 1986; Mundy et al., 1986; Dawson et al., 1998; Chawarska et al., 2013; Gangi et al., 2016), relatively few studies have explored this construct in children with FXS (Murphy et al., 2007; Hall et al., 2009; Hahn et al., 2016). Moreover, although autism symptoms are commonly observed in males with FXS, no studies directly compare JA performance across the two groups. The current study used an examiner-delivered task-based measure of JA skills in boys with autism and boys with FXS. When compared directly, no between-group differences in JA performance were observed. However, when considering boys of the same age, one must acknowledge that boys with autism typically demonstrate higher IQ scores and more autism symptomatology than boys with FXS. Taking this into consideration, we conducted comparisons controlling for the influences of age, non-verbal IQ, and autism symptom severity, and found that boys with FXS earned significantly higher JA scores than boys with autism. These data add to a growing body of research suggesting that, even though both boys with autism and boys with FXS demonstrate reduced gaze use in social interactions, a between-group difference may emerge between these two groups, particularly when other developmental factors are considered (Wolff et al., 2012; McDuffie et al., 2015).

Next, we considered the association between JA performance and language in boys with autism and boys with FXS. In boys with autism, JA performance was strongly associated with all language measures, even after controlling for non-verbal cognitive abilities. This finding is consistent with the extensive literature documenting the association of language abilities and JA performance in children with autism (Charman, 2003; Sigman and McGovern, 2005; Yoder et al., 2015; Bottema-Beutel, 2016). In contrast, the associations between JA performance and language were not significant in boys with FXS, after controlling for the influence of non-verbal cognitive ability and were weaker (*rs*: –0.19–0.20) than the associations observed for boys with autism (*rs*: 0.67–0.73). There is limited data considering the association between JA and language performance in FXS. Hahn and colleagues found that time spent in joint engagement states with caregivers (i.e., both the child and caregiver engaged with the same object) was positively associated with expressive language abilities (Hahn et al., 2016). Notably, joint engagement does not require the child to initiate JA; rather, the caregiver can support these states by following into the child’s attention to an object. These data suggest differences in the association between JA and language between boys with autism and boys with FXS. However, more research is needed to confirm these findings and to elucidate the nature of any differences and their contributions to language delays or any other similarities and differences observed between the autism and FXS phenotypes.

Lastly, we considered the associations between JA performance and other child characteristics such as autism symptom severity and other attention-modifying behavioral characteristics. Indeed, in addition to the link established between JA performance and autism (Mundy and Bullen, 2022), other factors such as inattention/hyperactivity and anxiety may also impact the development or measurement of JA (Leyfer et al., 2006; de Bruin et al., 2007; Cordeiro et al., 2011; Mayes et al., 2012; Thurman et al., 2014). These characteristics must be considered when comparing JA performance across conditions, such as autism and FXS, because the co-occurrence of these features likely differs across groups (McDuffie et al., 2013; Thurman et al., 2014, 2015). JA performance was associated with autism symptom severity for both groups. In addition, for boys, ADHD symptom severity was also associated with JA performance. Finally, for boys with FXS, anxiety symptom severity was significantly negatively associated with JA performance (*r* = –0.58); for boys with autism a non-significant positive correlation was observed between these variables (*r* = 0.27).

Data from the present study adds to the growing body of literature documenting an association between JA and both autism symptomatology and non-verbal cognitive development in autism and adds to the limited research considering JA skills in children with FXS (Sullivan et al., 2007; Constantino and Gruber, 2012; Lord et al., 2012; Brewe et al., 2018; Mundy and Bullen, 2022). Moreover, these findings not only point to potential underlying differences in factors contributing to JA performance in boys with autism and FXS, but also highlight the need for considering behavioral characteristics that may impact the development of JA in cross-syndrome research. Indeed, the co-occurring presence of attentional difficulties or anxiety can modify how children engage with others. Clarifying similarities and differences in the factors influencing JA will provide insight into when similar treatment or measurement approaches can be utilized when working with boys with autism or FXS and when phenotypic differences must be considered. For example, data from the present study suggests that the co-occurrence of anxiety in individuals with FXS may impact JA performance in this population. For boys with FXS, gaze avoidance has often been described to be related to anxiety. This may be attributable to higher rates of anxiety disorders, especially social anxiety, in FXS relative to the general population (Shaffer et al., 1996) and to other NDD groups (Dekker and Koot, 2003). Moreover, Thurman et al. (2014) previously observed maternal ratings of child social avoidance was related to child anxiety in FXS but not ASD. Taken together, it is posited that the co-occurrence of anxiety differentially impacts the measurement of JA in boys with autism and boys with FXS. More research is needed to disentangle the impact of anxiety from the association between JA and language development. For example, it may be that interactions with a more familiar partner, such as a caregiver, can provide additional insights into JA performance, and its role in language learning, in boys with FXS. Finally, ADHD symptomatology, not anxiety, was found to be associated with JA performance in boys with autism. Given the co-occurrence of ADHD and autism, it is necessary to better understand how ADHD symptomatology may impact the development of JA in boys with autism. Additional research elucidating the interrelations between these different domains may also provide insight into the developmental mechanisms underlying the autism phenotype.

Findings from the present study provide some important initial insights into potential similarities and differences in JA performance between boys with autism and boys with FXS. Nonetheless, there is much that remains to be understood. For example, in recent years, newer methods of considering joint attention have been developed and psychometrically validated (e.g., Nowell et al., 2020). It will be important for future studies comparing JA skills between individuals with autism and individuals with FXS to utilize a robust battery of JA measures, across different contexts, to ensure a thorough characterization of the similarities and differences observed across these groups. Moreover, larger longitudinal investigations of the associations between JA, language, and other phenotypic characteristics, are needed to more carefully consider findings suggested by the present study and to provide additional insights into potential cascading impacts of JA challenges on later skills across groups and to consider the intricate ways in which JA skills interact with other phenotypic characteristics (e.g., anxiety). Finally, because FXS is an X-linked disorder, and females with FXS have a second, unaffected X chromosome that can continue to serve as a protective factor, the present study focused on males only (Tassone et al., 1999; Loesch et al., 2004; Stembalska et al., 2016). It is vital that future research also consider JA performance in females with autism and females with FXS to consider whether findings in males also apply to females.

In summary, the present study compared JA performance between boys with autism and boys with FXS, as well as associations between JA, language and other child characteristics. Although overall JA performance was similar across the groups, JA scores were higher for boys with FXS when controlling for the influences of CA, non-verbal IQ, and autism symptom severity. In addition, potential between-group differences may emerge when considering the child characteristics associated with JA performance. Specifically, the positive association between JA performance and language was stronger in boys with autism than boys with FXS, after controlling for the influences of non-verbal IQ. In contrast, the negative association between JA performance and anxiety was stronger in boys with FXS than in boys with autism. These data suggest crucial differences in the contributors to JA performance, or the measurement thereof, and highlight the importance of considering whether similar or different developmental mechanisms underlie shared symptomatology between boys with autism and boys with FXS. Additional research is needed to elucidate the intricate associations between phenotypic features and JA is necessary to clarify the role of JA learning for boys with FXS and the extent to which differences in JA performance, and predictors thereof, contribute to the similarities and differences observed between the autism and FXS phenotypes.

## Data availability statement

The original contributions presented in the study are included in the article/supplementary material, further inquiries can be directed to the corresponding author/s.

## Ethics statement

The studies involving human participants were reviewed and approved by the University of California Davis Clinical Committee B. Written informed consent to participate in this study was provided by the participants’ legal guardian/next of kin.

## Author contributions

AT conceived of the study, participated in its design and coordination, performed the statistical analyses, and drafted the manuscript. AD helped design the analytic plan and draft the manuscript. Both authors read and approved the final manuscript.
